# Fabrication of Superhydrophobic Surfaces from Laser-Induced Graphene and Their Photothermally Driven Properties

**DOI:** 10.3390/ma18081880

**Published:** 2025-04-21

**Authors:** Yue Zhao, Yonghui Zhang, Yang Chen, Haodong Fu, Hao Liu, Jinlong Song, Xin Liu

**Affiliations:** State Key Laboratory of High-Performance Precision Manufacturing, Dalian University of Technology, Dalian 116024, China

**Keywords:** laser-induced graphene (LIG), low energy modification, Marangoni effect, photothermal conversion, micro actuator

## Abstract

Conventional LIG preparation mostly relies on the ablation process of a CO_2_ laser on a polyimide (PI) substrate but is limited by the sensitivity of the laser parameters, which is prone to PI film deformation, non-uniformity of the process, or LIG surface breakage problems. In this study, we present a new method to fabricate superhydrophobic laser-induced graphene (SH-LIG) surfaces by immobilizing the polyimide (PI) film on the copper sheet, which enables uniform laser processing (single pass laser etching) over a wider range of microsecond laser parameters (10.5–19.5 W). Subsequently, the SH-LIG was obtained by vacuum-assisted immersion in stearic acid, resulting in a water contact angle greater than 150°, roll angle stabilized at 6°, and hydrophobic stability at a high temperature of 90 °C. Analysis by Raman spectroscopy (Raman), X-ray photoelectron spectroscopy (XPS) and scanning electron microscopy (SEM) showed that the LIG fabricated at optimal power (19.5 W) had a more developed C sp^2^ network (I_2D_/I_G_ ≈ 0.5) and pore structure, which significantly improved the photothermal conversion efficiency (up to 252 °C in air and 180 °C on water). On this basis, a simple micro-driver based on SH-LIG was designed. Experiments showed that the maximum velocity of the SH-LIG boat can reach an adjustable propulsion velocity of 45.6 mm/s (related to the laser processing power and the intensity of the driving light), which is 132% higher than that of the LIG boat. This work provides insights into the preparation of high-quality LIG and their application in photothermally driven micro actuators, highlighting the synergies between structural optimization, surface engineering, and photothermal performance.

## 1. Introduction

In recent years, laser-induced graphene (LIG) has attracted much attention in the field of flexible electronics [1,2,3], sensors [4,5,6], energy harvesting [7,8], and energy conversion [9,10,11] due to its rapid fabrication [12,13], patterning [13,14], excellent conductive [15], and photothermal properties [16]. Conventional LIG preparation mostly relies on the ablation process of a CO_2_ laser on a polyimide (PI) substrate but is limited by the sensitivity of the laser parameters [17,18], which is prone to PI film deformation, non-uniformity of the process, or LIG surface breakage problems [19,20]. In addition, the highly hydrophilic surface of LIG limits its application in aqueous-based environments, especially in the field of actuation involving the Marangoni effect [20,21]. Whereas existing studies have mainly improved the hydrophobicity of LIG through complex plasma treatments or fluorination modifications [22,23], these methods suffer from drawbacks such as complex processes and environmental hazards. Although there are examples of direct superhydrophobicity of surfaces by controlling the laser parameters, they are only applicable to certain lasers and are not universally available [24]. And also, there is a lack of research on the use of a non-toxic, environmentally friendly, and low-cost low-surface-energy modifiers for hydrophobic treatment of LIGs. Moreover, the performance (e.g., velocity) of the Marangoni effect actuator is highly dependent on the photothermal conversion properties and surface wettability of the material [25], and the LIG-based Marangoni actuators reported so far are generally characterized by low driving velocity (<20 mm/s) [26]. Therefore, the development of superhydrophobic laser-induced graphene (SH-LIG) with a simple process and stable performance is of great scientific significance and application value.

In this paper, we present an innovative solution. By immobilizing the PI film with a copper sheet and 460 adhesives, the method allows the preparation of LIGs with uniform and unbroken surfaces stabilized by a microsecond infrared CO_2_ laser on LIGs with a wide range of powers, which is efficient, convenient, and cost effective. It does not require the use of high-cost lasers (e.g., femtosecond lasers) and avoids the problems of PI deformation caused by direct use of microsecond infrared lasers. The stearic acid vacuum-assisted immersion modification method was able to form a dense hydrophobic layer in the three-dimensional pore structure of LIG for simple and rapid preparation of SH-LIG. We have also systematically investigated the photothermal drive performance of LIG ships based on the Marangoni effect. The evolution of LIG morphology and chemical bonding mechanisms were revealed by SEM, XPS, and Raman spectroscopy, and the relationship between LIG quality and photothermal conversion performance was analyzed. It is demonstrated that the SH-LIG boat fabricated by this method can achieve a photothermal driving velocity of 45.6 mm/s, which is a significant improvement over the existing literature, and has excellent thermal cycle stability. This study not only provides a new method for the preparation of superhydrophobic laser-induced graphene, but also establishes a framework for designing smart light-responsive microsystems.

## 2. Materials and Methods

### 2.1. Materials

Anhydrous ethanol (analytically pure) and acetone (analytically pure) were purchased from Tianjin Damao Chemical Reagent Factory (Tianjin, China). Stearic acid (analytically pure) was purchased from Tianjin Chemical Reagent No. 3 Factory Co. (Tianjin, China). Commercial polyimide (PI) film (125 μm) was purchased from Shanghai East China Composite Insulation Filter Cloth and Screen Factory (Shanghai, China). Copper sheet (50 mm × 50 mm × 0.1 mm) was provided by Xinghua Jushun Hardware Manufacturing Factory (Xinghua, China). As well, 460 adhesive (Stickiness 1150.2000) was purchased from Dongguan Qingxi Polymerization Adhesive Products Manufacturing Factory (Dongguan, China).

### 2.2. Fabrication of SH-LIG

**Figure 1:**
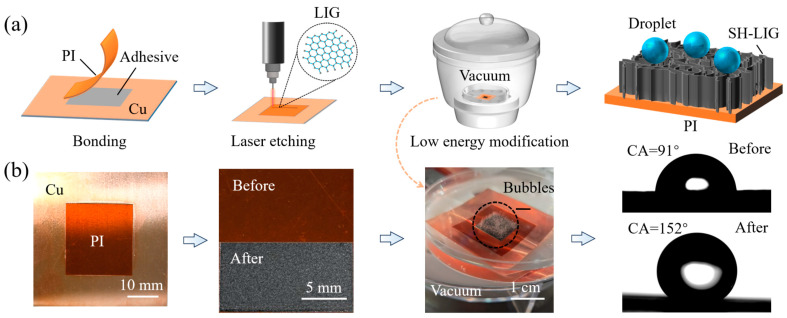
Fabrication process of SH-LIG. (**a**) Schematic diagram of SH-LIG fabrication process; (**b**) schematic diagram corresponding to the physical effect.

Figure 1a demonstrated the fabrication process of superhydrophobic laser-induced graphene (SH-LIG), where the PI film was cut to a square size of 25 × 25 mm, and washed sequentially with ethanol and deionized water for 5 min along with the copper sheet and then placed at room temperature for drying. Once drying was complete, the PI film was adhered to the center of the copper sheet with 460 adhesive and the air bubbles present between the PI film and the copper sheet were removed and the adhesive was left to cure for 5 min. A 10.6 μm CO_2_ microsecond laser (SK-CX30, Shanghai Sanke Laser Technology Co., Ltd., Shanghai, China) was used to etch along the grating once, with a grating width of 100 μm, an etching velocity of 200 mm/s, a frequency of 15 kHz, and a pulse width of 1 μs, and the PI film was located at the focal point (20.2 cm) of the laser for processing (details of the laser scanning overlap are shown in Figure S3). Due to the significant thermal effect of the CO_2_ laser process, the PI film suffered severe thermal deformation, which can be fixed on the copper sheet by #460 adhesive. Therefore, the PI film cannot suffer thermal deformation after laser etching, and high surface quality can be obtained. In addition, using copper sheet as the substrate with excellent thermal conductivity can increase the power window of laser processing. After our investigation, we found that due to the auxiliary fixation and heat dissipation of the copper sheet, the infrared CO_2_ laser in the range of 10.5–19.5 W can prepare uniform and unbroken LIGs on the surface of the PI film without penetrating the PI film. Moreover, the use of copper sheet to assist in the preparation of LIG does not cause the laser to penetrate the PI film, so the LIG is still present on the PI film. Therefore, the 460 adhesive between the PI film and the copper sheet can be cleaned off with acetone, and the LIG/PI composite layer can be obtained. The whole laser etching process was carried out under ambient conditions. Stearic acid and ethanol (mass ratio 0.018:1) were magnetically stirred at room temperature for 2 h and a mixture of 0.05 mol/L was configured. The SH-LIG was obtained by immersing the LIG in an ethanol stearate solution to form a solid coating. Figure 1b is the corresponding physical picture of Figure 1a, where it can be observed that a large number of bubbles are generated during the LIG vacuum treatment (Video S1).

### 2.3. Characterization

Contact angle and rolling angle were measured by an optical measurement instrument (SL200KS, Kino, Boston, MA, USA) with 8 μL of water droplet. The contact angle (CA) was measured based on the droplet reaching equilibrium on the surface, while the roll angle was recorded by rotating the substrate after the droplet had reached equilibrium until the droplet slid off. The contact angle and rolling angle were measured five times for each sample. A scanning electron microscope (SEM, JSM-7900F, Akishima, Japan) was used to observe the morphology of different microstructures. X-ray photoelectron spectroscopy (XPS, Thermo ESCALAB 250Xi, Waltham, MA, USA) and Fourier-transform infrared spectroscopy (FTIR, iS50, Waltham, MA, USA) were used to detect and analyze the chemical compositions of the HFE surfaces. X-ray photoelectron spectroscopy was performed with the Cls peak at 284.8 eV as a reference for all binding energies. Carbonization products at different laser fluxes were identified by using a Raman spectrometer with 532 nm (Raman, Invia Qontor, Renishaw, UK).

### 2.4. Photothermal Drive Experiment

The use of copper sheet to assist in the preparation of LIG does not cause the laser to penetrate the PI film, so the LIG is still present on the PI film. Therefore, the 460 adhesive between the PI film and the copper sheet can be cleaned off with acetone, and the LIG/PI composite layer can be obtained, as shown in Figure S4. After cleaning, LIGs could be obtained on both sides of the PI film LIGs and the LIGs were cut into isosceles triangles (initial dimensions: height 10 mm, base 8 mm) to serve as LIG boats. Placed in an acrylic sink filled with water, the LIG was illuminated with a driving light (LWIRPD-808-5F, Beijing Laser wave Optoelectronics Tech. Co., Ltd., Beijing, China) and moved in a straight line along the sink using the Marangoni effect. The driving light was placed on the guide driven by a stepper motor (adjustable velocity) (57HBP56AL4-TF8A, Beijing TIMES-CHAOQUN Electronic APPLIANCE Company, Beijing, China) and the sink (length 30 cm, width 3 cm, depth 3 cm) was parallel to the guide. As shown in Figure S6, in order to accurately measure the velocity of the LIG boat, we placed the LIG boat in an acrylic slot with a slot width of 2.5 cm. The rear part of the boat is irradiated by the driving light, which produces the Marangoni effect and moves the boat, but due to the limitation of the acrylic slot (the length of the acrylic slot is 30 cm and the height is 3 cm), the boat can only keep moving forward. As shown in Figure S7, the drive light source is placed on the guide rail of the stepper motor, the stepper motor can drive the drive light source to move in a straight line on the guide rail. The stepper motor controller can adjust the speed of the stepper motor to control the velocity of the drive light source on the guide rail. At the same time, the linear motion of the drive light source is kept parallel to the linear motion of the LIG ship (by constantly adjusting the relative positions of the two). The stepper motor controller then indicates the velocity of the LIG boat when the two remain relatively stationary during linear motion (with the drive light source always illuminating the stern of the LIG boat). Lines 129–135 of the revision add details of the method of measuring the velocity of the boat (Figure S5, Video S2). The LIG boat with their hydrophobic surfaces facing downward gained the velocity of the SH-LIG boat, and those with their hydrophobic surfaces facing upward gained the velocity of the LIG boat. The velocity of the LIG boat can be controlled by laser processing power (initial parameter: 19.5 W), driving light intensity (initial parameter: 2.5 A), and boat size (scaled down based on the initial size). When exploring the effect of a single parameter on the driving performance of the LIG boat, the parameters of the other two were constant.

## 3. Results and Discussion

**Figure 2:**
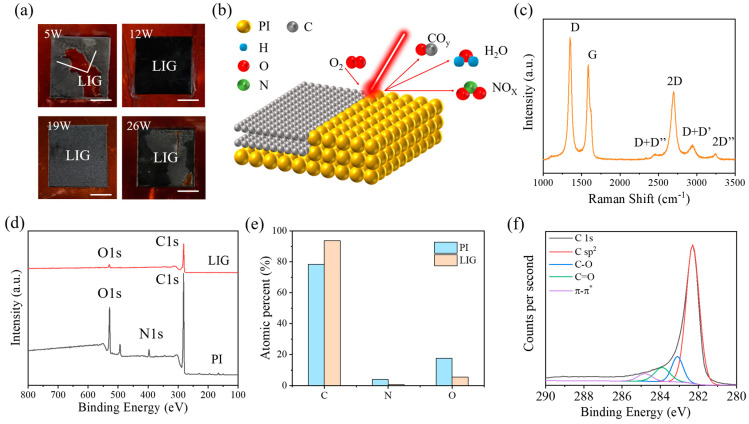
Laser-etched PI surface and its chemical composition analysis. (**a**) Optical images of LIG surfaces by laser etching with power of 5 W, 12 W, 19 W, and 26 W (scale bar: 5 mm); (**b**) schematic diagram of laser-etched PI film; (**c**) Raman spectra of laser-etched PI surfaces; (**d**) comparison of X-ray photoelectron spectroscopy (XPS) results of PI film and LIG; (**e**) the atomic percentage of PI and LIG; (**f**) C1s spectra of PI.

To explore a suitable laser power for LIG preparation, we first selected powers with 5 W, 12 W, 19 W, and 26 W in the full power range (0–30 W) of the CO_2_ microsecond laser to laser etching the PI film (scanning only once), as shown in Figure 2a. It can be found that the four laser powers can fabricate the black material on the PI film, which was then performed by Raman spectroscopy, as shown in Figure 2c. The results of the Raman plot showed a typical Raman shift of the LIG, similar to reduced graphene oxides (rGOs) [27]. The D band located near 1300 cm^−1^ exhibited a sharp peak, indicating many defects in the material, and the G band located near 1580 cm^−1^ represented the content of C sp^2^, which was the main form of C atoms in graphene. Finally, the distinct peak located at 2500–2800 cm^−1^ was the 2D band, which usually indicated the presence of graphene and its derivatives. The sharper the peak, the higher the graphene content, which was the fingerprint signal of graphene [28,29,30]. In contrast, the D + D′, D + D″ and 2D′ bands appearing around the 2D peaks were formed by complementary formation of defects in graphene materials, such as rGOs [27].

To further investigate the chemical properties of LIG, we performed X-ray photoelectron spectroscopy (XPS) of LIG and compared it to the unprocessed PI film as a control, as shown in Figure 2d. In the XPS results of LIG and PI film, LIG showed a substantial increase in C1s intensity compared to PI film, while O1s and N1s decreased substantially. Further quantitative analysis of the elemental content of C, N, and O (Figure 2e) revealed that the C content of the laser-treated LIG is higher than that of the PI, and correspondingly, the oxygen content in the LIG is reduced from 17.62% with respect to the PI film to 5.57%. The XPS results (Figure 2d) and elemental content analysis (Figure 2e) indicated the denitrification and deoxygenation process during LIG preparation. In addition, deconvolution of the C1s peaks in the XPS map of LIG into four peaks centered at 284.8 (C-C), 285.3 (C-O-C), 286.1 (O=C-N), and 287.0 eV (O=C-O), combined with the previous Raman spectra (Figure 2c), illustrated that the major carbon elements consisted of C sp^2^ and the residual C-O and C=O (Figure 2f) [30]. Therefore, we speculated that during laser processing of PI to form LIG, laser irradiation of the PI surface causes the temperature of the PI surface to rise dramatically and breaks the chemical bonds (C=O, C=C and C-N) of the PI molecules. At this time, the carbon atoms in PI reorganize into the carbon skeleton of graphene, while other elements such as N and O generate gases. The expansion of the gases creates an impact on the surrounding area, resulting in the formation of the loose and porous structure of LIG, as shown in Figure 2b. From Figure 2a, it can be seen that we can fabricate LIG by a wide range of laser power based on our method, but there are some defects on the LIG surface prepared at some laser powers, such as laser etching inhomogeneity at 5 W power and LIG surface breakage at 26 W power, which led to an inability to follow up the investigations on the Marangoni effect. To obtain the optimal laser etching power that can be processed uniformly and the surface will not be broken, we further explored the laser processing power. Finally, it was found that the laser power in the interval of 10.5–19.5 W can fabricate uniform LIG on the surface of the PI film, which is called the effective power range (EPR). Seven specific powers (10.5 W, 12 W, 13.5 W, 15 W, 16.5 W, 18 W, and 19.5 W) were selected in the effective power interval, which is called effective power (EP).

**Figure 3:**
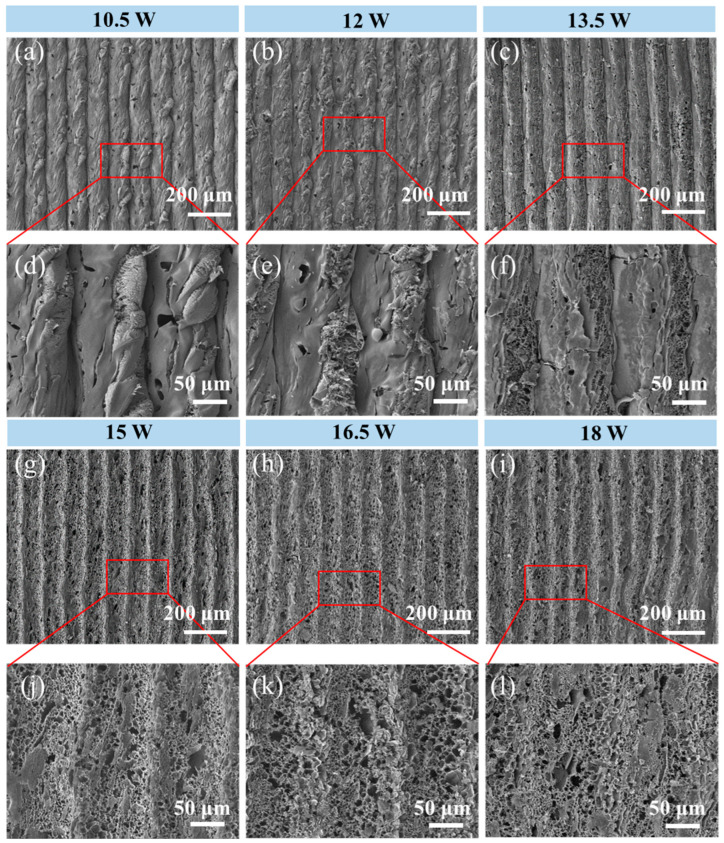
SEM images of LIG fabricated by different effective powers (EP). (**a**) SEM image of LIG fabricated by 10.5 W; (**b**) SEM image of LIG fabricated by 12 W; (**c**) SEM image of LIG fabricated by 13.5 W; (**d**) local enlargement of SEM image of 10.5 W; (**e**) local enlargement of SEM image of 12 W; (**f**) SEM image of localized LIG surface fabricated by 13.5 W; (**g**) SEM image of LIG fabricated by 15 W; (**h**) SEM image of LIG fabricated by 16.5 W; (**i**) SEM image of LIG fabricated by 18 W; (**j**) SEM image of localized LIG surface fabricated by 15 W; (**k**) SEM image of localized LIG surface fabricated by 16.5 W; (**l**) SEM image of localized LIG surface fabricated by 18 W.

**Figure 4:**
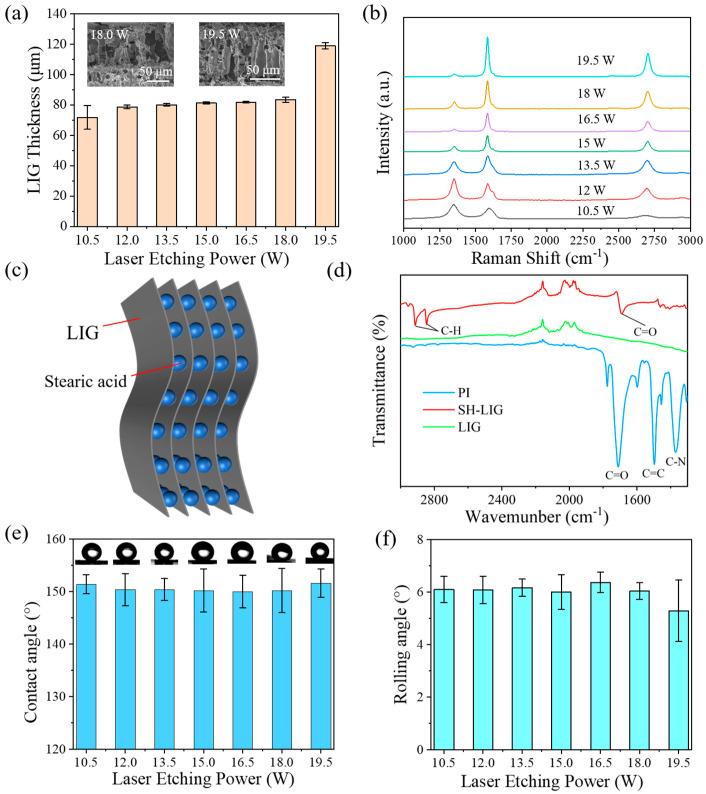
(**a**) Thickness of LIG at different laser etching powers (EP); (**b**) Raman spectra of LIG at different laser etching powers (EP); (**c**) schematic diagram of SH-LIG hydrophobic principle; (**d**) comparison of FTIR spectrum of PI, LIG, and SH-LIG; (**e**) contact angle of SH-LIG with different laser etching powers (EP); (**f**) rolling angle of SH-LIG with different laser etching powers.

Figure 3a–l and Figure S2 show the SEM characterization of EP-fabricated LIG, and the surface morphology of the LIG showed a clear regularity with the increase in the laser etching power. Firstly, it could be clearly seen that the laser etching path of the laser on the PI film had a width of roughly 0.1 mm, which coincided with our set laser parameters. Secondly, the laser etching path gradually faded with increasing power, and completely disappeared when the power was 19.5 W (Figure S2), but then the pore structure on the LIG surface increased. Figure 4a and Figure S3 show the thickness statistics and cross-sectional SEM characterization of the EP-fabricated LIG. The cross-sectional morphology of the LIG was shaped as if the trees were grown on a PI film, and their thickness and porous structure increased with increasing laser power. In addition, Raman spectra of EP-fabricated LIG (Figure 4b), where *I*_2D_/*I*_G_ allowed a rough estimation of the number of layers of graphene, was performed, with single-layer graphene > 2, bilayer ≈ 1, and tetra-layer ≈ 0.5 in the Raman spectrum of classical graphene [31,32]. The LIG fabricated at 19.5 W power can reach *I*_2D_/*I*_G_ ≈ 0.5, which is the highest among all EPs, and the intensity of the D peak is also the lowest, indicating that the LIG fabricated at 19.5 W power possesses the best quality, and that 19.5 W is the best power among EPs with the number of graphene layers close to four layers. Combined with the LIG formation mechanism we summarized before (Figure 2b), it could be assumed that the gases (NO_x_, CO_y_) formed during the formation of LIG have impacted the surrounding materials, which would prompt the graphene network to grow upward and form porous structures. The high laser etching power of 19.5 W allowed the reaction to proceed more completely, and thus the LIG with a higher thickness and more sparse and porous structures could be obtained.

The large number of loose and porous structures possessed by LIG was the key to obtaining SH-LIG using a stearic acid vacuum-assisted immersion method, as shown in Figure 4c. Stearic acid can effectively bond with the micro- and nanostructures on the surface of graphene, while the vacuum treatment was able to draw out the air between the LIG pores, during which a large number of bubbles could be observed (Figure 1b and Video S1). The stearic acid solution was also able to penetrate deeper into the porous structure of the LIG, enhancing the uniformity of the stearic acid coating, whereas, under vacuum conditions, the accelerated volatilization of ethanol from the solution and the accelerated precipitation of stearic acid solutes reduces its agglomeration during crystallization and forms a denser hydrophobic layer, as shown in Figure 4c. The chemical groups on the surface of PI, LIG, and SH-LIG were analyzed using FTIR spectroscopy (Figure 4d). It can be seen that the characteristic peaks of C=O, C=C, and C-N on the surface of the laser-processed samples have all disappeared when compared with the initial PI; and the surface modified by stearic acid has reappeared with the characteristic peaks of C=O again. Compared to the FTIR spectra of LIG, the absorption peaks in the high-frequency region of the FTIR spectra of SH-LIG were located near 2913 cm^−1^ and 2846 cm^−1^, which belong to the asymmetric and symmetric stretching vibrations of C-H, respectively. In the low-frequency region, an absorption peak of 1697 cm^−1^ appeared on the surface of SH-LIG compared to LIG, which belonged to the stretching vibration of C=O in the carboxyl group [33]. Considering the chemical modifications, the appearance of the C peak proved the presence and bonding of CH_3_(CH_2_)_16_COO^−^ on the SH-LIG surface. Comparison of the wettability of SH-LIG fabricated by EP in Figure 4e,f shows that all the contact angles could be achieved up to ~150° and the rolling angle could be stabilized at about 6°, which demonstrated the stability and universality of the stearic acid vacuum-assisted immersion method.

**Figure 5:**
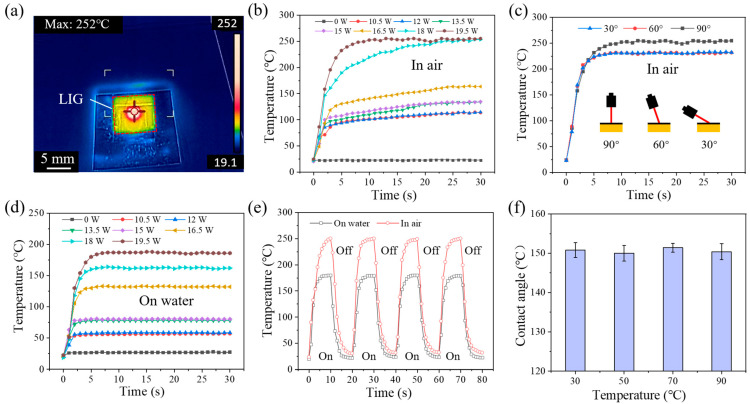
Photothermal performance of LIG. (**a**) Infrared thermography of LIG prepared in air with 19.5 W laser etching power; (**b**) variation in LIG temperature with time under the same light conditions in air; (**c**) variation in LIG (19.5 W) temperature with time for different light incidence angles; (**d**) variation in LIG temperature with time under the same light conditions on water; (**e**) variation in LIG temperature with time under intermittent illumination; (**f**) variation in contact angle with temperature for SH-LIG.

Materials with high photothermal conversion efficiency are a prerequisite for fabricating Marangoni effect actuators, so it is important to investigate the photothermal conversion properties of LIG. Figure 5a shows the maximum temperature (252 °C) reached by a 19.5 W power-fabricated LIG with a 2.5 A driving light. Figure 5b shows the variation in LIG temperature in air with time for LIG fabricated with different EP powers and raw PI (0 W) under the same light conditions (driving light, 2.5 A). It could be seen that the temperature of LIG surfaces fabricated by EP powers could reach 100 °C in air, while the temperature of the PI film as a control group was almost unchanged, and the temperature of LIG fabricated with 19.5 W power even reached 250 °C. Considering the practical application scenario of LIG, the LIG was placed on the water, as shown in Figure 5d, and it could be found that the rate of temperature increase with time and the maximum achievable temperature of each power have decreased, but the maximum could be still reached to 180 °C. Figure 5c shows the variation in LIG temperature with time for LIG (19.5 W) with the laser at different incidence angles (90°, 60°, 30°). In the same irradiation time, the rate of increase of LIG temperature was basically the same, and although there was a difference in the maximum temperature of the three, there was only a difference of about 20 °C (less than 10%), so it could be concluded that the laser incidence angle had almost no effect on the LIG temperature. We then conducted intermittent light experiments (in air and on water) on LIG (19.5 W), as shown in Figure 5e. Intermittent light experiments with 10 s of light on (intensity 2.5 A) and 10 s of light off were performed on the LIG, showing that the LIG has excellent photothermal cycling ability. Considering the possibility that the hydrophobic coating of stearic acid might fail at high temperatures, it was necessary to conduct thermal stability experiments on the coating, as shown in Figure 5f, which shows that the wettability of the coating was basically unchanged after being subjected to different temperatures for a period of time.

In summary, LIG demonstrated excellent light-to-heat conversion and thermal cycling capabilities both in air and on water. Moreover, the hydrophobic LIG coating maintained excellent thermal stability after a period of high temperature. The LIG fabricated at 19.5 W shows the best photothermal conversion performance. Combining the previous results of Raman spectra (Figure 4), XPS characterization (Figure 2f), and SEM (Figure S3), we could speculate that the photothermal conversion ability of the LIG was related to the SP^2^-type carbon content in the network of LIG carbon atoms (height of the G-peak) and its formation of a porous three-dimensional structure. The high level of defects (*I*_D_/*I*_G_) in LIG then disrupted the conjugate structure of C sp^2^, corroborating the above observation. As shown in Table S1, we know that as the laser etching power increases, the LIG thickness increases. The two properties of Raman spectra, *I*_D_/*I*_G_, which represent the defect content, decrease gradually with power, and reach the lowest in the preparation power of 19.5 W, which is only 0.081, implying that at this time the C sp^2^ network in the LIG is extremely well developed and the content is extremely high. On the other hand, *I*_2D_/*I*_G_, which represents the information of the number of layers in LIG, increases with the increase in laser etching power, indicating that the average number of layers in LIG is decreasing in the power range of 10.5–19.5 W, and its nature is closer to that of monolayer graphene. Finally, it is reflected in the photothermal conversion performance, and the LIG prepared at 19.5 W reached the highest temperature of 255 °C.

**Figure 6:**
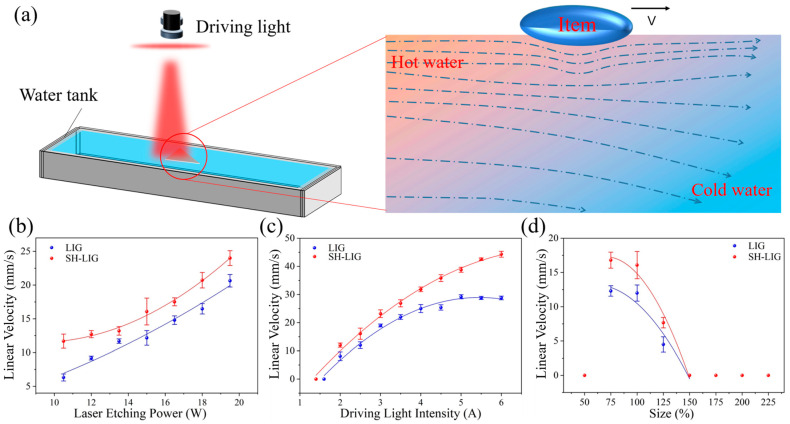
Application of LIG based on Marangoni effect. (**a**) Schematic diagram of the principle of the Marangoni effect that drives the movement of the LIG boat; (**b**) velocity of LIG and SH-LIG boats at different laser etching powers; (**c**) velocity of LIG and SH-LIG boats under different driving light intensities; (**d**) variation in LIG and SH-LIG boat velocity with boat size.

Based on the excellent photothermal and hydrophobic properties of the SH-LIG, a micro actuator with high velocity can be achieved. Figure 6a shows a schematic diagram of the Marangoni effect of the LIG boat in the water, which was driven by the light source irradiation. At the stage, a large amount of heat could be generated and dissipated into the water. The water temperature near the LIG boat was rising locally, resulting in a certain temperature difference, which altered the balance of the surface tension of the water. At this point, water would flow from the low surface tension region (high temperature) to the high surface tension region (low temperature), which would drive the LIG boat, as shown in Figure 6b. The maximum velocity of the LIG boat may be affected by laser etching power, light intensity, and size of the actuator. First, we explored the relationship between the laser etching power and the maximum velocity of the LIG boat, as shown in Figure 6c. The velocity of the SH-LIG boat is higher than that of the LIG boat, and the velocities of the two boats increase with the increase in the laser etching power. The SH-LIG boat achieves a maximum velocity of 25.2 mm/s compared to the LIG boat. Combined with the results shown in Figure 5d and Table S1, this can be attributed to the fact that the LIG has higher quality and lower defects with increasing laser etching power, which results in the LIG having a stronger photothermal conversion efficiency at higher preparation power. Figure 6c shows the relationship between the driving light intensity and the velocity of the LIG boat, and the velocity of the SH-LIG boat also exceeds that of the LIG boat. The LIG boat can reach 19.6 mm/s, while the maximum velocity of the SH-LIG boat can reach 45.6 mm/s, which is 132% faster than that of the LIG boat. This can be attributed to the fact that the higher the intensity of the driving light, the more the light energy is converted by the LIG, resulting in a larger temperature difference at the surface of the water, which produces a more pronounced Marangoni effect, and therefore, an increase in maximum velocity as the intensity of the light source increases. The main reason for the subsequent gradual slowing down of the trend in increasing velocity of the boat is that when the intensity of the driving light is too high, it will lead to an overall increase in the water temperature, which will lead to a re-equilibrium of the water temperature difference. It was also worth noting that the SH-LIG boat was capable of starting tiny movements with a driving light intensity of 1.4 A, compared to 1.6 A for LIG boat. Therefore, the superhydrophobic coating helps to reduce the drag on the forward motion of the LIG boat. Figure 6d shows the relationship between the size of the LIG boat and the maximum velocity, and both the LIG small boat and the SH-LIG small boat can only move at 75%, 100%, and 125% of the size, implying that the size of the LIG boat is either too small or too large to produce a Marangoni effect drive. The reason for this is that when the boat is too small, the heat generated by the photothermal conversion is too small to generate enough of a temperature difference to produce the Marangoni effect [34]. When the boat is too large, the resistance of the water has exceeded the driving force that can be generated by the Marangoni effect, and therefore, motion is also not possible.

## 4. Conclusions

In conclusion, we developed a method based on vacuum-assisted stearic acid-modified superhydrophobic laser-induced graphene (SH-LIG) and realized its efficient application in photothermal drive based on the Marangoni effect. The LIGs can be processed in a larger laser power range (10.5–19.5 W) and the number of LIG layers fabricated at the optimal power (19.5 W) is close to four by means of the copper sheet fixation and 460 adhesive fixation method. The vacuum-assisted immersion modification of stearic acid formed a dense coating within the pores of the LIG, allowing the LIG to maintain hydrophobic stability at higher temperatures (90 °C). The LIG demonstrates excellent photothermal conversion (250 °C in air, 180 °C on water) and photothermal cycling capabilities. For photothermal drive, the SH-LIG boat can reach a maximum velocity of 45.6 mm/s, 132% faster than the LIG boat, and at the same time, have a lower drive start-up threshold light intensity (1.4 A). These results break through the power interval limitation and surface modification bottleneck of traditional LIG fabrication, and the developed SH-LIG Marangoni actuator shows a broad application prospect in the fields of environmental monitoring and microfluidic manipulation. Future work will focus on the complex motion trajectory control of SH-LIG boats to further expand its potential for applications in the field of self-drive.

## Data Availability

The original contributions presented in this study are included in the article/supplementary material. Further inquiries can be directed to the corresponding authors.

## Supplementary Materials

The following supporting information can be downloaded at https://www.mdpi.com/article/10.3390/ma18081880/s1, Figure S1: SEM image of LIG (19.5 W); Figure S2: SEM image of cross section of LIG (19.5 W); Figure S3: Details on the overlap between laser scans; Figure S4: Schematic diagram of copper sheet-assisted LIG preparation; Figure S5: Schematic diagram of a boat driven by a driving light source.; Figure S6: Schematic diagram of the movement of the LIG boat in an acrylic tank; Figure S7: Schematic diagram of the velocity measurement method for the LIG small boat; Table S1: List of LIG properties; Video S1: Stearic acid vacuum-assisted immersion treatment; Video S2: LIG Small boat photothermal drive experiment.
