# Supplementary material for: Fabrication of Superhydrophobic Surfaces from Laser-Induced Graphene and Their Photothermally Driven Properties
Source: Materials (Basel). 2025 Apr 21;18(8):1880. doi: 10.3390/ma18081880 (PMC12028950; doi:10.3390/ma18081880)
Supplement: Supplementary file 1 [file materials-18-01880-s001.zip › Supplementary Information.docx]

Supplementary Information for

Fabrication of superhydrophobic surfaces from laser-induced graphene and their photothermally driven properties

Yue Zhao^‡^, Yonghui Zhang^‡^, Yang Chen, Haodong Fu, Hao Liu, Jinlong Song* and Xin Liu*

State Key Laboratory of High-performance Precision Manufacturing, Dalian University of Technology, Dalian 116024, P. R. China

***** Correspondence: [songjinlong@dlut.edu.cn](mailto:songjinlong@dlut.edu.cn) (J.S.); [xinliu@dlut.edu.cn](mailto:xinliu@dlut.edu.cn) (X.L.)

**^‡^** These authors contributed equally to this work.

**Content**

**Figure S1.** SEM surface image of LIG fabricated by 19.5 W.

**Figure S2.** SEM cross-section of LIG fabricated at 19.5 W.

**Figure S3.** Details on the overlap between laser scans.

**Figure S4.** Schematic diagram of copper sheet-assisted LIG preparation.

**Figure S5.** Schematic diagram of a boat driven by a driving light source.

**Figure S6.** Schematic diagram of the movement of the LIG boat in an acrylic tank.

**Figure S7.** Schematic diagram of the velocity measurement method for the LIG small boat.

**Table S1.** List of LIG properties.

**Video S1.** Stearic acid vacuum-assisted immersion method.

**Video S2.** LIG-based small boat speed test.


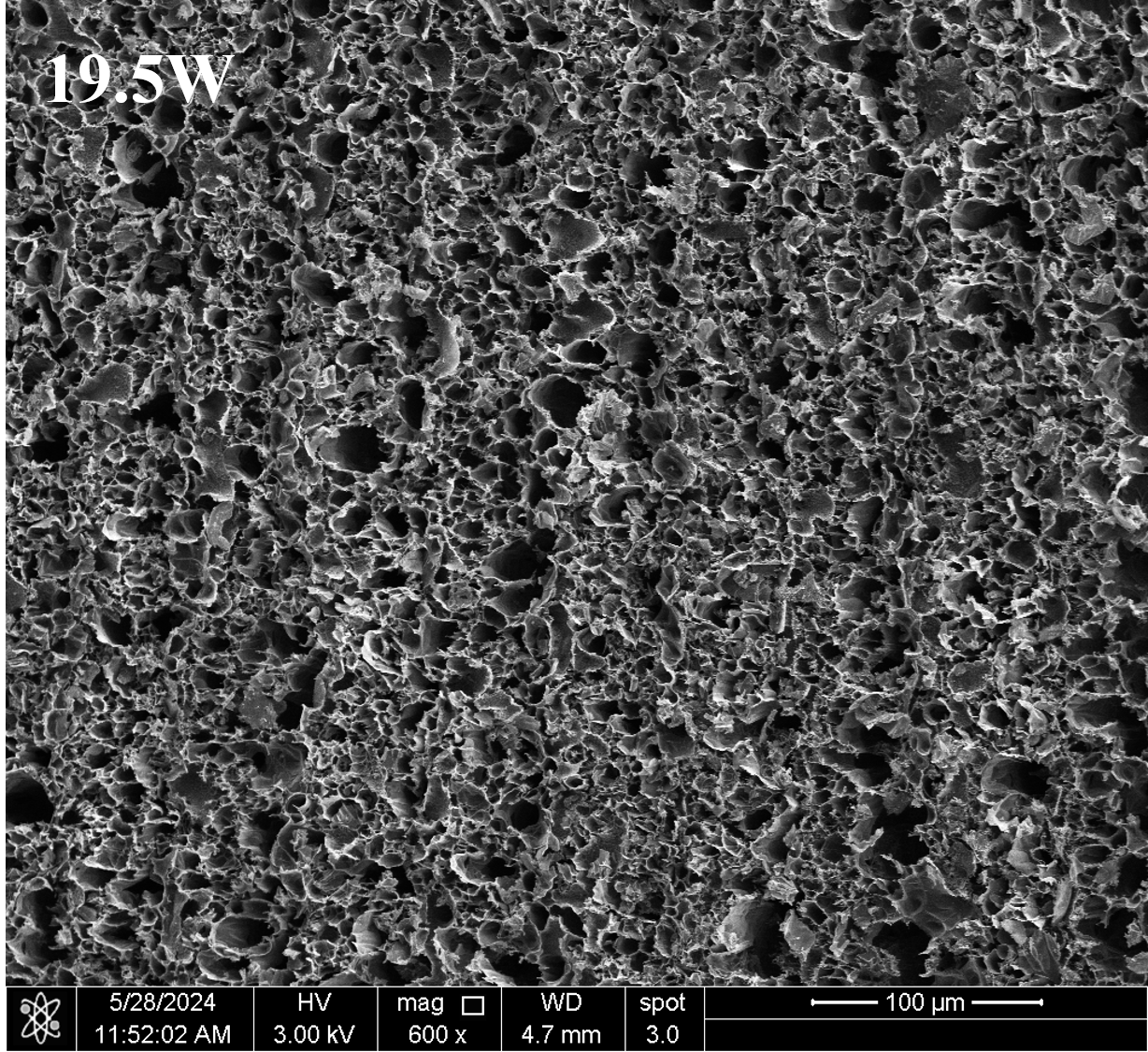


**Figure S1.** SEM surface image of LIG fabricated by 19.5 W.


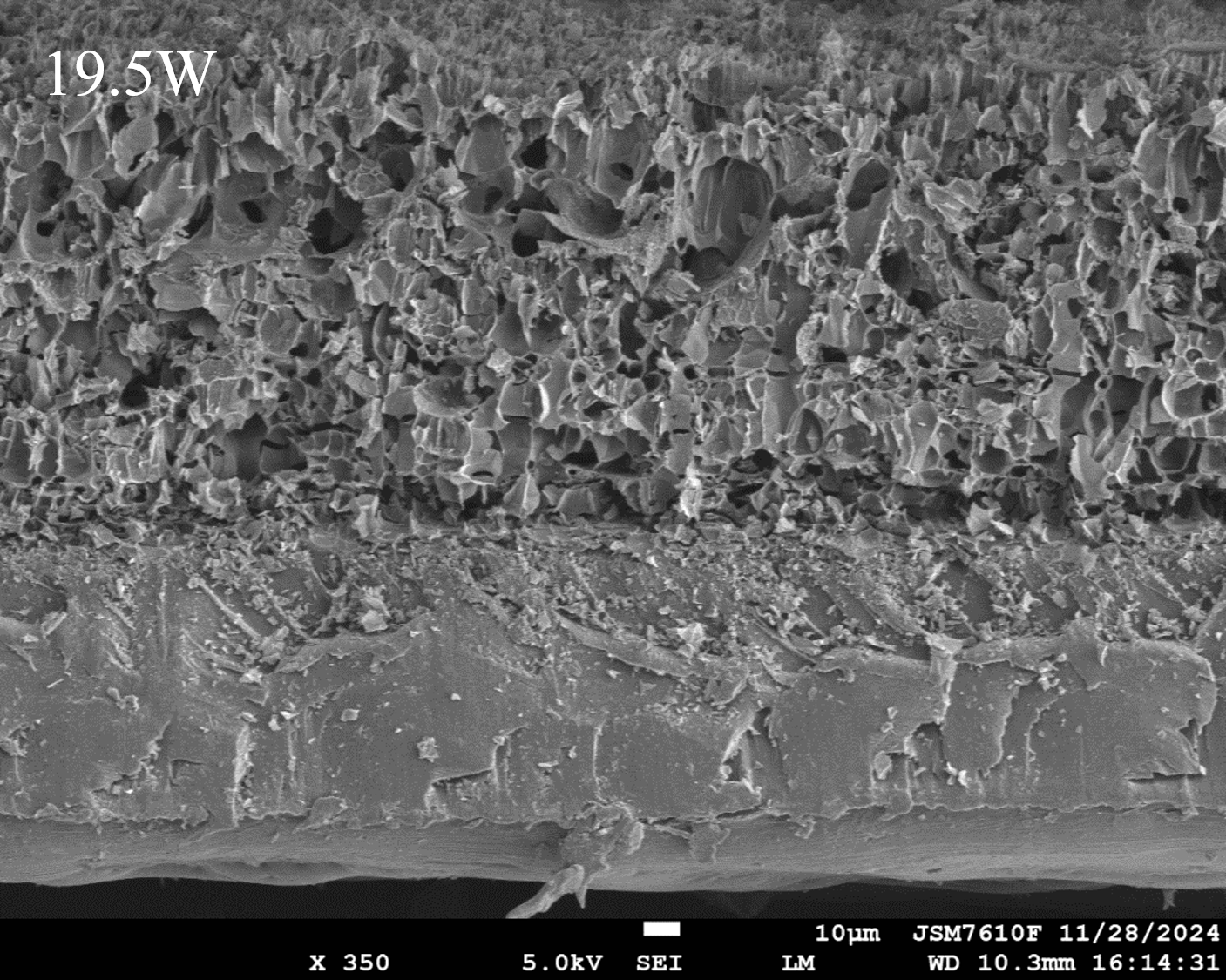


**Figure S2.** SEM cross-section of LIG fabricated at 19.5 W.

**
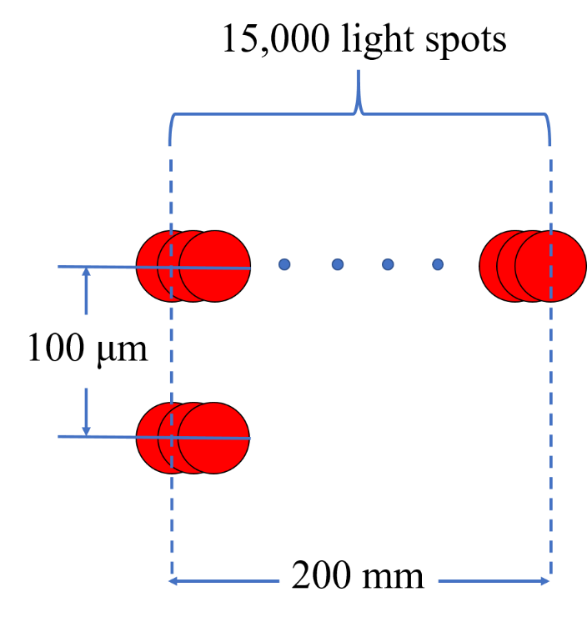
**

**Figure S3.** Details on the overlap between laser scans.

**
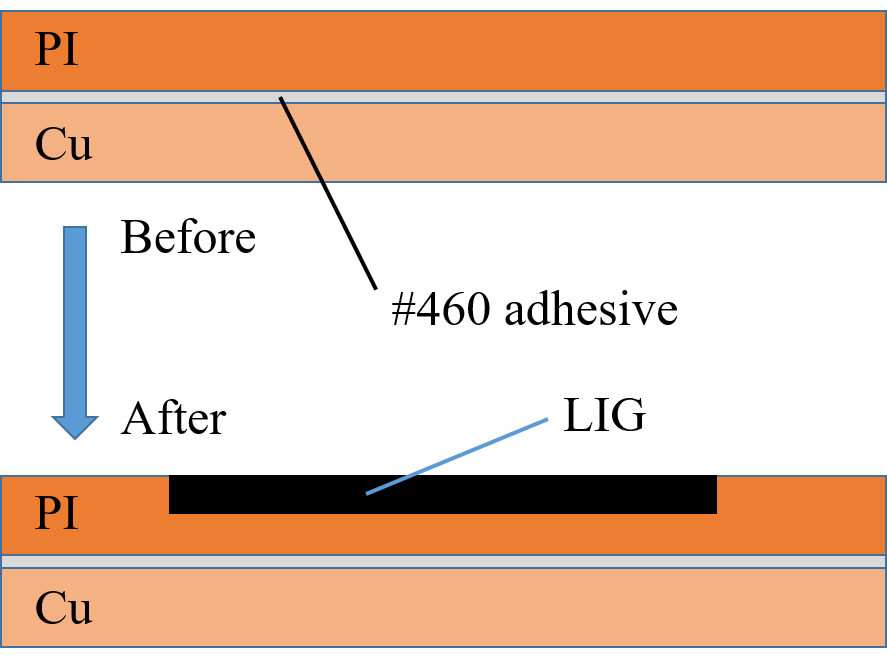
**

**Figure S4.** Schematic diagram of copper sheet-assisted LIG preparation.


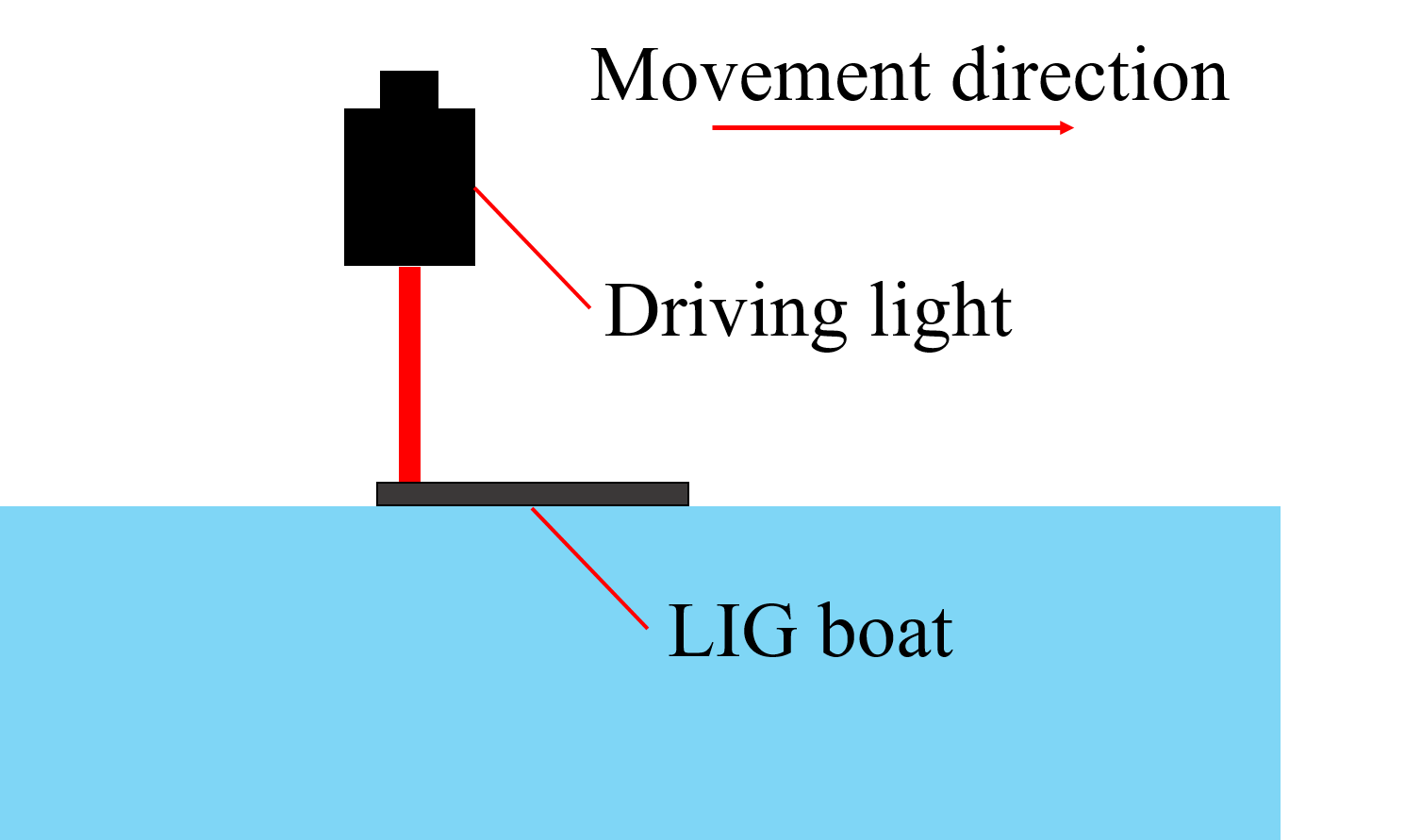


**Figure S5.** Schematic diagram of a boat driven by a driving light source.


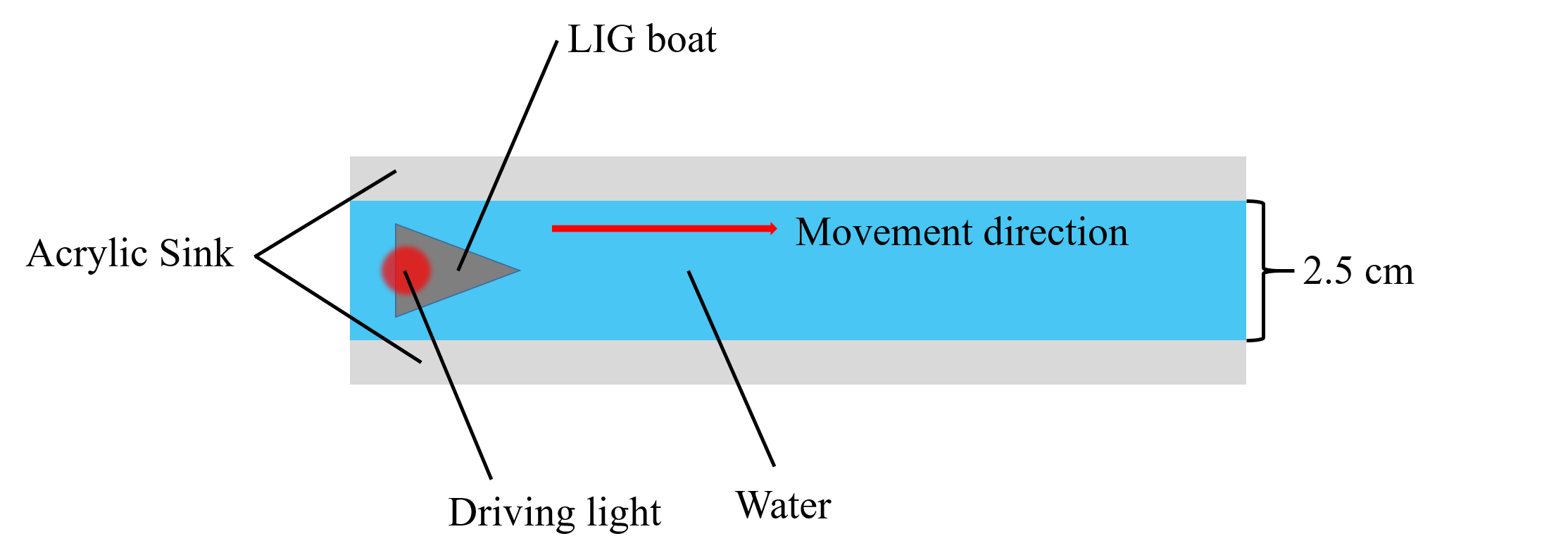


**Figure S6.** Schematic diagram of the movement of the LIG boat in an acrylic tank.


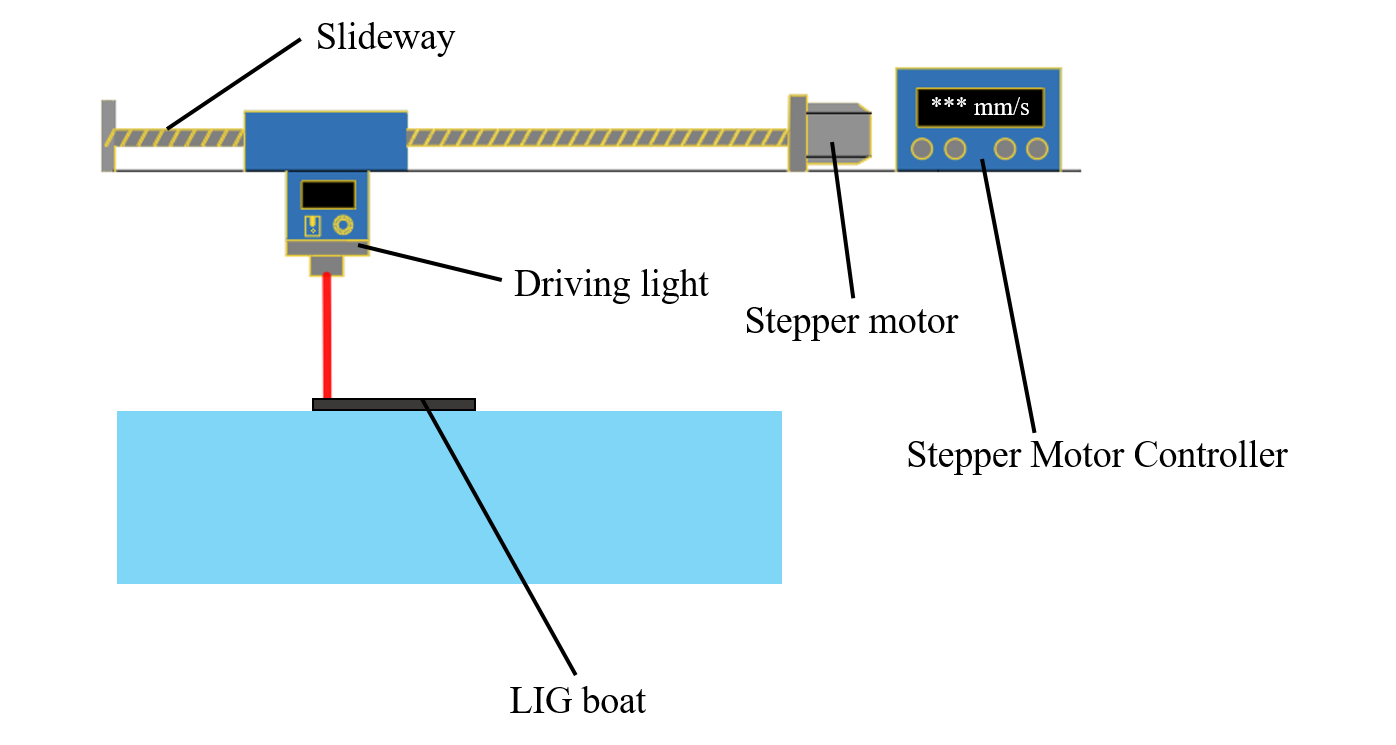


**Figure S7.** Schematic diagram of the velocity measurement method for the LIG small boat.

**Table S1.** List of LIG properties.

| **Laser etching power** | **LIG thickness** | **Raman (***I*_D_/*I*_G_**)** | **Raman (***I_2_*_D_/*I*_G_**)** | **Highest temperature** |
| --- | --- | --- | --- | --- |
| 10.5 W | 71.86 μm | 1.335 | 0.362 | 113 ℃ |
| 12 W | 78.78 μm | 1.275 | 0.73 | 114 ℃ |
| 13.5 W | 80.16 μm | 0.682 | 0.765 | 133 ℃ |
| 15 W | 81.36 μm | 0.341 | 0.658 | 134 ℃ |
| 16.5 W | 81.84 μm | 0.139 | 0.635 | 163 ℃ |
| 18 W | 83.48 μm | 0.267 | 0.596 | 253 ℃ |
| 19.5 W | 108.92 μm | 0.081 | 0.563 | 255 ℃ |
